# Reclassifying No Specific Molecular Profile Endometrial Cancer: A Narrative Review of Molecular Heterogeneity, Prognostic Refinement, and Clinical Management

**DOI:** 10.3390/cancers18193223

**Published:** 2026-10-07

**Authors:** Yujing You, Yan Zhang

**Affiliations:** 1 Department of Obstetrics and Gynecology, Peking University First Hospital, Peking University Health Science Center, Beijing 100034, China; 2411210238@stu.pku.edu.cn; 2 Department of Obstetrics and Gynecology, Peking University First Hospital, Beijing 100034, China

**Keywords:** endometrial cancer, no specific molecular profile, heterogeneity, prognostic biomarker, clinical management

## Abstract

Molecular classification has transformed the management of endometrial cancer (EC), yet the no specific molecular profile (NSMP) subtype remains a major challenge for precision oncology due to its substantial biological and clinical heterogeneity. This narrative review summarizes evidence on the molecular features, candidate prognostic biomarkers, procedural implications, and treatment data relevant to NSMP EC and proposes a conceptual framework for further study. Emerging evidence indicates that NSMP represents a heterogeneous disease spectrum rather than a uniform entity. Histological grade and estrogen receptor (ER) expression provide important prognostic refinement, while molecular alterations, including *CTNNB1* mutations, *ARID1A* abnormalities, and L1CAM overexpression, may further discriminate risk. Current trials and guidelines increasingly support biomarker-driven treatment strategies, although prospective validation is needed. In conclusion, NSMP EC requires further refinement beyond conventional risk classification. We propose an evidence-informed three-layer conceptual framework integrating molecular classification (*POLE*, MMR, and p53 status), clinicopathological factors (grade and ER expression), and emerging biomarkers, including *ARID1A* and L1CAM. This framework is intended to organize current evidence and generate hypotheses for future validation, which may facilitate individualized prognostic assessment of NSMP EC.

## Simple Summary

No specific molecular profile endometrial cancer is the largest molecular group of endometrial cancers, but patients within this group can have markedly different outcomes. This review examines the clinical and biological factors that may explain this variation. Tumor stage, histological type and grade, lymphovascular space invasion, and estrogen-receptor expression currently provide the most clinically useful prognostic information. Other features, including CTNNB1 and ARID1A alterations, L1CAM expression, and spatial immune phenotypes, may further refine risk, but their clinical value remains unconfirmed. We propose a three-layer conceptual framework that separates established molecular classification from clinicopathological risk assessment and exploratory biomarkers. This framework is intended to propose hypotheses for risk re-stratification of NSMP tumors based on existing evidence and guide future research, not to serve as a treatment algorithm. Prospective studies are required before emerging biomarkers can be used routinely to select surgery, adjuvant therapy, or systemic treatment.

## 1. Introduction

Endometrial cancer is the most common gynecologic malignancy in many high-income settings, and its incidence is increasing [1]. In 2013, The Cancer Genome Atlas (TCGA) described four molecular groups—*POLE*-ultramutated, microsatellite instability-hypermutated, copy-number-low, and copy-number-high tumors—through integrated genomic analysis [2]. Clinically applicable surrogate classifiers, including the Proactive Molecular Risk Classifier for Endometrial Cancer (ProMisE) and the TransPORTEC approach, subsequently enabled assignment to *POLE*-mutated (*POLE*mut), mismatch repair-deficient (MMRd), p53-abnormal (p53abn), and NSMP groups using targeted sequencing and immunohistochemistry (IHC) [3,4]. NSMP is assigned by exclusion of the other three classifiers and overlaps substantially, but not completely, with the TCGA copy-number-low group. Although NSMP has an intermediate prognosis at the group level, outcomes vary according to stage, histotype, grade, lymphovascular space invasion (LVSI), hormone-receptor expression, and additional molecular features. Furthermore, we propose an evidence-informed three-layer conceptual framework to organize established and emerging prognostic factors within NSMP EC. Rather than constituting a validated clinical model, this framework is intended to provide a structured synthesis of current evidence and to highlight priorities for future prospective validation.

## 2. Methods

For this narrative review, PubMed/MEDLINE was searched for English-language publications from 1 January 2020 to 27 September 2026. A reproducible example of the search strategy was: (“endometrial cancer”[Title/Abstract]) AND (“NSMP”[Title/Abstract] OR “no specific molecular profile”[Title/Abstract] OR “ProMisE”[Title/Abstract] OR “molecular classification”[Title/Abstract]) AND (“estrogen receptor”[Title/Abstract] OR “CTNNB1”[Title/Abstract] OR “ARID1A”[Title/Abstract] OR “L1CAM”[Title/Abstract] OR “immune phenotype”[Title/Abstract] OR “surgical staging”[Title/Abstract] OR “treatment”[Title/Abstract]). Seminal earlier studies, current international guidelines, and additional records identified from reference lists were also considered.

Studies were selected according to their relevance to NSMP-specific biological, prognostic, or therapeutic questions. When multiple publications addressed the same topic, priority was given to current international guidelines, randomized or prospective trials, large molecularly annotated cohorts, and systematic reviews or meta-analyses. For multiple reports from the same trial or cohort, the most recent and complete publication was preferentially cited, while earlier reports were retained when they provided distinct analyses or outcomes not available in the later publication. Because this was a narrative review, study selection and synthesis were qualitative; no protocol registration, formal risk-of-bias assessment, meta-analysis, or statistical derivation of the proposed framework was undertaken.

## 3. Heterogeneous Prognosis of NSMP Endometrial Cancer

NSMP EC has conventionally been described as an intermediate-prognosis molecular group, with outcomes that are generally worse than those of *POLE*mut disease and better than those of p53abn disease [3]. This group-level description, however, does not capture the full range of clinical behavior. In a cohort of 167 non-endometrioid endometrial carcinomas, NSMP tumors represented 27% of cases and had 3-year recurrence-free survival (RFS) and overall survival (OS) rates of 73.3% and 82.2%, respectively [5]. Diagnostic discordance between p53 IHC and *TP53* status can also affect surrogate classification in a minority of tumors [6,7]. In a small series of 56 clear cell carcinomas, molecular subgrouping offered limited prognostic separation, whereas advanced stage or metastatic disease was the dominant adverse factor [8]. Conversely, among 367 stage I–III high-grade endometrioid or non-endometrioid carcinomas staged by lymphadenectomy without adjuvant therapy, the NSMP subgroup had a 5-year recurrence rate of 37.9% and an OS rate of 55.7%, similar to the corresponding estimates in the p53abn group [9]. These findings support the use of clinicopathologic context when interpreting NSMP status; they do not imply that all NSMP tumors share a uniformly high or low risk.

## 4. Mechanisms Underlying the Heterogeneity of NSMP Endometrial Cancer

### 4.1. Divergent Genomic Landscapes

Genomic studies have identified recurrent alterations in *PTEN*, *PIK3R1*, *PIK3CA*, *KRAS*, and other genes within NSMP EC [10]. Analysis of early-stage, non-myoinvasive NSMP tumors suggested several evolutionary patterns, including clonal *PTEN* alterations with *PIK3CA*/*PIK3R1*/*ARID1A* co-alterations, concurrent *PIK3CA* and *ARID1A* changes, *KRAS* mutation with 1q gain, and isolated *AKT1* mutation [11]. These data indicate that NSMP is not a single genomic entity. The proposed subclasses are descriptive, however, and have not yet been shown to define treatment-responsive groups.

### 4.2. Immune Microenvironment Heterogeneity

NSMP endometrial carcinomas show substantial variation in the spatial distribution of tumor-associated immune cells. Based on the localization of CD8-positive tumor-infiltrating lymphocytes, tumors can be categorized as inflamed, immune-excluded, or immune-desert types. In two cohorts totaling 145 endometrial carcinomas, Hattori et al. identified 65 NSMP tumors; 66.2% had an immune-desert phenotype, whereas 13.8% were inflamed [12]. RNA-sequencing analyses suggested enrichment of type I interferon-response genes in non-inflamed NSMP tumors, although these molecular findings were exploratory. These observations establish heterogeneity in immune-cell localization within NSMP disease but do not demonstrate treatment-predictive value.

## 5. Risk Re-Stratification Strategies for NSMP Endometrial Cancer

### 5.1. Sex-Hormone Receptor Expression Status

Large molecularly annotated cohorts support ER expression, interpreted together with tumor grade and other clinicopathologic factors, as a prognostic refiner within NSMP EC. In a cohort of 1110 NSMP tumors, Jamieson et al. defined a lower-risk subgroup as grade 1–2 and ER-positive (>1% tumor-cell IHC staining); this group had a 5-year disease-specific mortality of 1.6%, compared with 22.9% among grade 3 and/or ER-negative tumors (HR, 16.3; 95% CI, 8.4–31.7) [13]. In a separate analysis of 648 high-risk ECs, including 202 NSMP tumors, Vermij et al. found that ER positivity (≥10%) was independently associated with a lower risk of recurrence within the NSMP subgroup (HR, 0.33; 95% CI, 0.15–0.75). The 5-year RFS was 80.9% for ER-positive and 45.3% for ER-negative NSMP tumors [14]. Although a 10% cutoff showed greater prognostic discrimination than a 1% cutoff within that cohort, this exploratory comparison does not establish a universally applicable threshold. ER IHC may therefore contribute to prognostic refinement in NSMP EC, but assay methods and cutoff definitions should be reported explicitly, and ER status should not be used alone as a treatment-predictive biomarker or treatment-selection rule.

Evidence is less definitive in high-grade NSMP tumors. Timmermans et al. evaluated 57 high-grade NSMP carcinomas and reported 5-year OS estimates of 46% for ER-negative and 65% for ER-positive disease; the difference did not reach statistical significance (*p* = 0.068) [15]. The cohort was small and the direction of effect remained compatible with a clinically relevant difference. These data therefore do not establish that ER lacks prognostic value in high-grade disease, nor do they justify considering all high-grade NSMP tumors biologically uniform.

The 2025 ESGO–ESTRO–ESP guidelines distinguish NSMP low-grade/ER-positive tumors (ER expression ≥ 10%) from NSMP high-grade and/or ER-negative tumors (ER expression < 10%). Final prognostic risk-group assignment, however, depends on integrating this subdivision with FIGO 2023 stage, tumor extent, and LVSI status. The guidelines define estimated overall 5-year recurrence risks of <8%, 8–14%, 15–24%, and ≥25% for the low-, intermediate-, high–intermediate-, and high-risk groups, respectively [16].

Progesterone receptor (PR) is commonly co-expressed with ER. In a meta-analysis of 4654 patients with NSMP EC, PR positivity was associated with lower hazards of recurrence (HR, 0.37; 95% CI, 0.25–0.57) and death (HR, 0.26; 95% CI, 0.13–0.52) [17]. However, PR did not consistently retain independent significance after adjustment for ER and grade in individual cohorts [13]. PR is therefore best interpreted as an adjunct to ER and grade rather than as a standalone clinical classifier.

### 5.2. CTNNB1 Mutations

Exon 3 mutations in *CTNNB1* impair β-catenin degradation and activate Wnt signaling [18]. These mutations have been associated with recurrence in early-stage, low-grade endometrioid carcinomas [18,19]. A meta-analysis of seven studies including 1031 patients reported an association with recurrence in early-stage disease (OR, 3.00; *p* = 0.019) and in analyses restricted to NSMP tumors (HR, 2.83; *p* = 0.026) [20]. The magnitude of risk may depend on co-alterations and clinicopathologic features [21]. In a 125-patient NSMP cohort, De Leo et al. reported that *CTNNB1*-altered/*ARID1A*-wild-type tumors had no observed recurrences, whereas concurrent *ARID1A* alterations were associated with higher grade, necrosis, tumor budding, and worse outcomes [22]. This interaction requires confirmation in independent cohorts.

The international, multicenter, randomized phase III PORTEC-4a trial prospectively evaluated a molecular-integrated profile–guided strategy for adjuvant treatment in patients with high-intermediate-risk endometrial cancer [23]. Within this integrated framework, NSMP tumors with wild-type *CTNNB1* were included in the favorable molecular profile and were assigned to observation, whereas NSMP tumors harboring *CTNNB1* exon 3 mutations were classified as an intermediate molecular profile and received vaginal brachytherapy. The molecular-profile–guided strategy met the predefined criterion for non-inferiority with respect to vaginal recurrence compared with standard vaginal brachytherapy. These findings support the feasibility of incorporating *CTNNB1* status into a multivariable molecular-integrated treatment strategy. However, they do not establish *CTNNB1* status as an independently predictive biomarker for benefit from, or omission of, adjuvant therapy. In particular, because treatment allocation differed according to the integrated molecular profile, the independent effect of *CTNNB1* mutation within NSMP tumors cannot be isolated from the trial design. Further prospective studies are therefore needed to determine whether *CTNNB1* provides treatment-predictive information beyond its potential prognostic value.

### 5.3. ARID1A Mutations

*ARID1A* encodes a core subunit of the SWI/SNF chromatin-remodeling complex and is frequently altered in NSMP endometrial carcinoma, but its prognostic value remains unsettled. In a retrospective cohort of 72 NSMP tumors, including 24 with *ARID1A* mutations, Onoprienko et al. reported higher recurrence risk and shorter progression-free survival (PFS) after multivariable adjustment (OR, 3.70; 95% CI, 1.04–13.13; and HR, 3.19; 95% CI, 1.10–9.25, respectively), without a significant association with disease-specific survival [24]. In a Chinese cohort of 151 NSMP tumors, Yuan et al. identified *ARID1A* mutations in 74 tumors (49.0%) and reported shorter PFS (*p* = 0.019). Associations appeared stronger in tumors with *PIK3CA* hotspot co-mutations or ER/PR double negativity, although these subgroup findings were exploratory [25].

Pooled evidence is inconsistent. Marchetti et al. reported an adverse PFS association limited to NSMP tumors (HR, 2.35; 95% CI, 1.09–5.06) [26], whereas a separate systematic review and meta-analysis by Ferrari et al. found no significant pooled association of *ARID1A* mutations with recurrence or survival [17]. In the Yuan cohort, mutation status was associated with protein loss (*p* < 0.001), but 64.4% of mutation-positive tumors retained *ARID1A* expression, and IHC status itself was not significantly associated with PFS [25]. Thus, mutation status and protein expression should not be regarded as interchangeable prognostic measures.

### 5.4. L1CAM Overexpression

Elevated L1 cell adhesion molecule (*L1CAM*) overexpression (>10%) is associated with increased tumor invasiveness, metastatic dissemination, and unfavorable clinical outcomes [17]. In 112 patients with NSMP tumors, L1CAM expression above 10% was associated with shorter time to recurrence (HR, 2.64; 95% CI, 1.06–6.56; *p* = 0.037) and higher cancer-specific mortality (HR, 3.87; 95% CI, 1.44–10.40; *p* = 0.007) [27]. In a separate single-center cohort of 79 NSMP tumors, L1CAM positivity was present in 15.2% and was associated with higher recurrence (41.7% vs. 7.5%, *p* = 0.001) and lower 5-year OS (58.3% vs. 88.1%, *p* = 0.002) and PFS (58.3% vs. 92.5%, *p* = 0.001) [28]. Furthermore, a recent meta-analysis synthesizing available evidence proposes a pragmatic dichotomy: low-grade NSMP tumors with ER positivity and L1CAM expression ≤10% may be classified as low-risk, whereas high-grade tumors or those exhibiting ER negativity or L1CAM overexpression (>10%) warrant classification as high-risk NSMP [26]. Collectively, these retrospective associations support further evaluation of L1CAM as a prognostic refiner.

From a practical perspective, both L1CAM and *ARID1A* can be evaluated in formalin-fixed, paraffin-embedded specimens using standard immunohistochemical platforms, making them technically accessible and potentially less resource-intensive than sequencing-based assays. However, broader implementation will require standardized scoring criteria, particularly for the threshold of L1CAM positivity and the interpretation of heterogeneous or partial *ARID1A* loss. Although IHC-based assessment may reduce testing burden, its incremental clinical utility and cost-effectiveness beyond established clinicomolecular classification remain to be demonstrated.

### 5.5. Immune Phenotypes and Markers of Immune Evasion

Retrospective studies suggest that spatial immune phenotypes may provide prognostic information beyond the NSMP designation. In the 145-case series reported by Hattori et al., the inflamed phenotype was associated with longer progression-free survival and overall survival than the non-inflamed phenotypes (*p* = 0.004 and *p* = 0.005, respectively), with a similar pattern reported in the NSMP subset; however, NSMP-specific adjusted effect estimates were not provided. De Biase et al. independently classified 213 endometrial carcinomas, including 86 NSMP tumors, using whole-section immunohistochemistry and digital image analysis. In the NSMP subgroup, 3-year disease-free survival was 96.2% (95% CI, 75.7–99.5%) for the inflamed phenotype, 83.2% (95% CI, 60.7–93.5%) for the desert phenotype, and 40.5% (95% CI, 10.0–70.1%) for the excluded phenotype (log-rank *p* < 0.001; median follow-up, 23 months) [29]. An association was also observed among adjuvant-treated NSMP cases, but the study did not test interaction with treatment and therefore does not establish predictive utility.

HLA class I (HLA-I) loss represents a related marker of immune escape. In a retrospective series, it was associated with recurrence in stage I–II NSMP disease (adjusted HR, 24.63; 95% CI, 2.40–257.27), although the wide confidence interval and limited number of events indicate substantial uncertainty [30]. These findings are hypothesis-generating and require external validation before clinical application.

Collectively, spatial immune phenotypes and HLA-I loss may provide additional prognostic information within NSMP disease, but their independent clinical utility requires validation in larger, treatment-annotated cohorts.

## 6. An Evidence-Informed Framework for Prognostic Refinement

Current evidence supports a hierarchical interpretation of prognostic factors in NSMP endometrial carcinoma. Histological grade, ER expression, FIGO stage, and lymphovascular space invasion have the strongest current clinical support for prognostic refinement. In addition, *CTNNB1* mutations and L1CAM overexpression have been associated with adverse outcomes. Notably, both them have been incorporated as an unfavorable prognostic marker into the integrated molecular risk-stratification algorithm evaluated in prospective trials such as PORTEC-4a. By contrast, the prognostic significance of alterations in *KRAS*, *ARID1A*, and other genes remains inconsistent across existing studies. These biomarkers are therefore still considered exploratory, and should not currently be used as routine stratification criteria for adjuvant treatment decision-making.

To distinguish routinely applicable factors from exploratory biomarkers, the relative evidence levels and current clinical roles of established and emerging prognostic refiners in NSMP endometrial carcinoma are summarized in Table 1.

**Figure 1:**
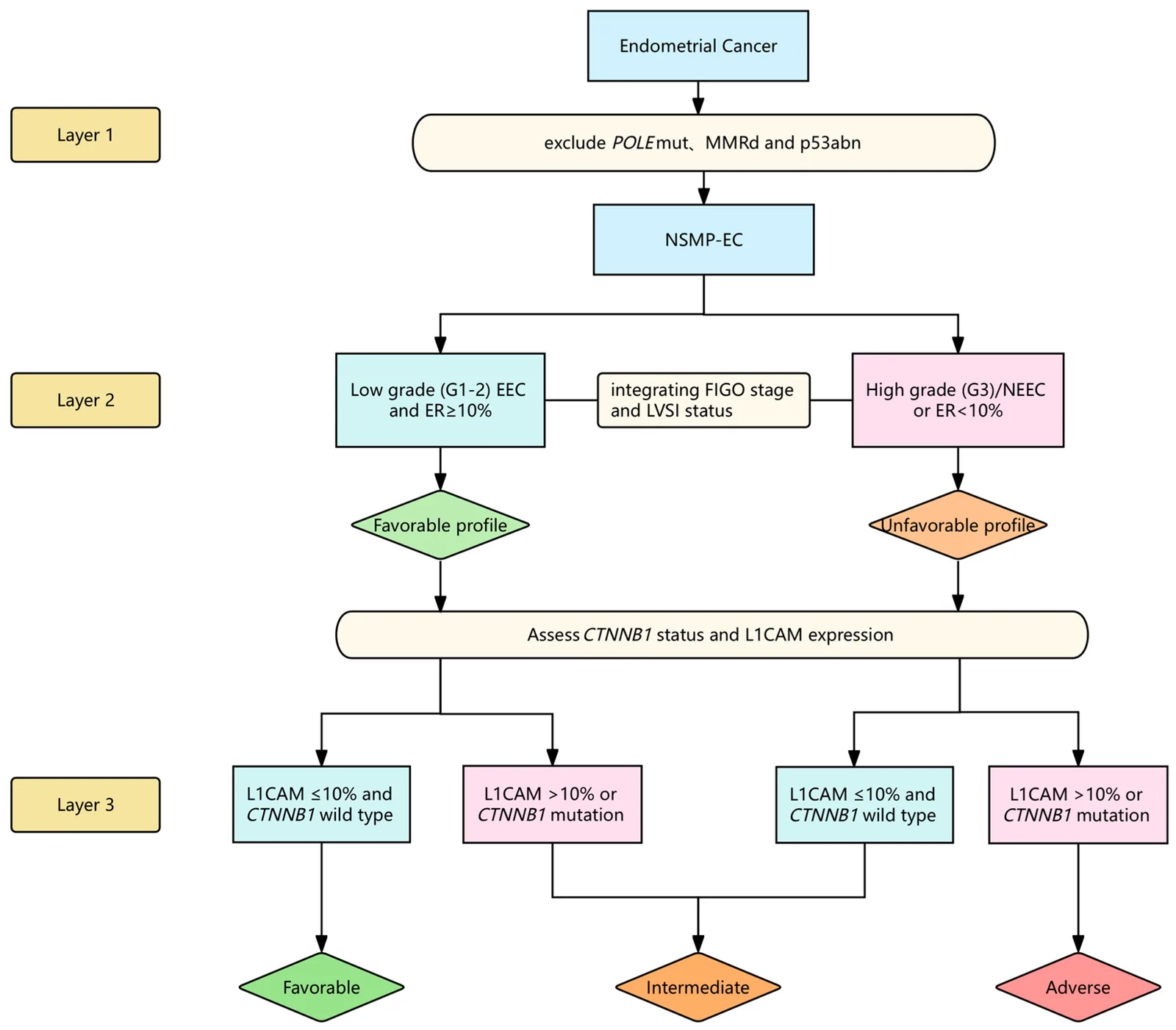
An Evidence-Informed Three-Layer Conceptual Framework for Prognostic Refinement in NSMP Endometrial Cancer. Abbreviations: NSMP-EC, no specific molecular profile endometrial cancer; EEC, endometrioid endometrial carcinoma; NEEC, non-endometrioid endometrial carcinoma; ER, estrogen receptor.

Based on the available evidence summarized in this review, we propose an evidence-informed three-layer conceptual framework for organizing prognostic information in NSMP endometrial carcinoma (Figure 1). This framework is intended to illustrate how established and emerging factors may be integrated conceptually rather than to serve as a formally validated risk-prediction algorithm.

The first tier applies the established molecular classifier based on pathogenic *POLE* exonuclease-domain mutations, mismatch repair status, and p53 status to identify true NSMP tumors. The second tier characterizes NSMP carcinomas according to clinicopathological features as having either a favorable profile, defined by low-grade (G1–G2) endometrioid histology and ER positivity (IHC ≥ 10%), or an unfavorable profile, defined by high-grade (G3) and/or non-endometrioid histology and/or ER negativity (IHC < 10%). These designations represent clinicopathological profiles rather than complete clinical risk categories. Final guideline-based risk assignment requires integration with FIGO 2023 stage and LVSI status. The third layer incorporates emerging biomarkers, including L1CAM overexpression and *CTNNB1* alterations, as exploratory prognostic refiners.

However, this framework should be viewed as a hypothesis-generating structure designed to organize existing evidence and provide a basis for future prospective studies. Prospective validation and external validation are required before it can be used to guide decisions regarding adjuvant therapy.

## 7. Clinical Management in NSMP Endometrial Cancer

The clinical implications of NSMP molecular heterogeneity differ across surgical, adjuvant, and advanced-disease settings and should therefore be considered separately.

### 7.1. Procedural Implications: Preoperative Molecular Classification and Surgical Staging

Molecular classification may also have implications for preoperative risk assessment and surgical staging. In a cohort of 172 patients undergoing lymph-node staging, molecular subtype was significantly associated with lymph-node metastasis, which was detected in 10.8% of NSMP tumors compared with 44.8% of p53-abnormal tumors [31]. This association was further supported by the multicenter SENECA study of 2139 patients with early-stage endometrial cancer, in which sentinel lymph-node involvement occurred in 7.8% of NSMP tumors, compared with 12.5% and 12.4% of p53-abnormal and MMR-deficient tumors, respectively [32].

For molecular information to influence surgical planning, reliable classification on preoperative biopsy specimens is essential. Recent matched biopsy–hysterectomy studies have demonstrated high concordance of molecular classification, supporting its technical feasibility before definitive surgery. Nevertheless, the residual risk of nodal involvement in NSMP tumors remains clinically relevant, and molecular subtype alone is insufficient to justify omission of nodal staging. Consistently, the 2025 ESGO–ESTRO–ESP guidelines continue to recommend sentinel lymph-node biopsy as the preferred nodal-staging procedure for presumed early-stage endometrial cancer. Thus, molecular classification may refine preoperative risk assessment but should currently complement, rather than replace, established surgical staging criteria.

### 7.2. Adjuvant Therapy of Early-Stage NSMP Disease

The 2025 ESGO/ESTRO/ESP guidelines provide more refined recommendations for adjuvant treatment of NSMP endometrial carcinoma by integrating the 2023 FIGO stage, lymphovascular space invasion, histological grade, and ER status [16]. Nevertheless, the magnitude of benefit derived from radiotherapy and chemotherapy in this molecular subgroup remains to be further clarified in NSMP-specific clinical trials. Molecular subgroup analysis of the PORTEC-3 trial showed that, although the 5-year recurrence-free survival rate was numerically higher among patients with NSMP tumors receiving chemoradiotherapy than among those receiving radiotherapy alone (80% vs. 68%), the difference was not statistically significant [33]. At 10 years, no OS benefit was observed in the ER-positive NSMP subgroup (81.8% vs. 82.3%). The ER-negative subgroup included only six patients allocated to chemoradiotherapy and seven allocated to radiotherapy, precluding a reliable treatment-effect estimate [34]. Given that the incidence of grade ≥3 toxicity in the overall PORTEC-3 population was substantially higher with chemoradiotherapy than with radiotherapy alone (61% vs. 13%), adjuvant endocrine therapy, such as maintenance progestin therapy, may represent a less toxic alternative for patients with ER-positive NSMP tumors. This strategy is currently being evaluated in the randomized phase III NSMP-ORANGE trial within the RAINBO program (NCT05255653) [35].

### 7.3. Systemic Treatment of Advanced or Recurrent NSMP Endometrial Cancer

#### 7.3.1. Chemoimmunotherapy with or Without ICI/PARP Inhibitor Maintenance

Carboplatin–paclitaxel remains a common chemotherapy backbone for advanced or recurrent EC, with immune checkpoint inhibitors (ICI) incorporated into first-line regimens on the basis of randomized trials in molecularly broad populations. NSMP-specific estimates are generally exploratory subgroup analyses and should be interpreted accordingly.

The phase III RUBY trial demonstrated that dostarlimab plus chemotherapy followed by dostarlimab maintenance reduced the risk of disease progression by 23% in the NSMP subgroup compared with chemotherapy alone (HR, 0.77; 95% CI, 0.55–1.07), although the confidence interval included unity [36]. Thus, while the findings are consistent with potential activity in NSMP disease, they do not establish a definitive NSMP-specific predictive effect.

Building on this, RUBY Part 2 evaluated dostarlimab plus chemotherapy followed by maintenance therapy with dostarlimab and niraparib. In the initial PFS analysis reported in 2024, a prespecified exploratory analysis showed a nominal PFS signal in the NSMP subgroup (HR, 0.61; 95% CI, 0.38–0.99) [37]. However, the subsequent full report published in 2026 did not demonstrate a clear OS benefit in this subgroup (HR, 0.91; 95% CI, 0.50–1.68). In the overall safety population, grade ≥3 treatment-related adverse events occurred in 70.7% versus 37.5% of patients, and treatment discontinuation because of adverse events occurred in 38.7% versus 11.5%, respectively [38]. Thus, the PFS finding in NSMP remains hypothesis-generating, and the benefit–risk profile of PARP inhibitor–containing maintenance therapy has not been established for molecularly confirmed NSMP disease.

The phase III DUO-E trial evaluated durvalumab plus chemotherapy followed by maintenance durvalumab with or without olaparib [39]. In the pMMR/*TP53*-wild-type subgroup, durvalumab plus olaparib maintenance reduced the risk of progression or death by 43% compared with chemotherapy alone (HR, 0.57; 95% CI, 0.44–0.73), with a median PFS of 15.0 months; the corresponding estimates for durvalumab maintenance alone were an HR of 0.77 and a median PFS of 9.9 months. Importantly, the pMMR/*TP53*-wild-type definition may enrich for NSMP disease but does not exclude all *POLE*mut or multiple-classifier tumors and is not equivalent to confirmed NSMP. Consequently, the DUO-E findings provide only indirect, hypothesis-generating support for PARP inhibitor–containing maintenance strategies.

#### 7.3.2. Endocrine Therapy Combined with CDK4/6 Inhibition

Single-arm phase II studies suggest activity of endocrine therapy combined with CDK4/6 inhibition in ER-positive advanced or recurrent EC. Fulvestrant plus abemaciclib produced an overall objective response rate (ORR) of 44% among 25 response-evaluable patients; the ORR was 59% in the copy-number-low/NSMP subgroup and 13% in other molecular subgroups [40]. Letrozole plus abemaciclib produced an ORR of 30% and median PFS of 9.1 months in 30 patients with ER-positive recurrent EC, 73% of whom had NSMP disease [41]. Responses observed in small biomarker-defined subsets, including tumors with *CTNNB1* mutations, are hypothesis-generating rather than evidence of a predictive biomarker.

Building on evidence that metformin can delay therapeutic resistance via the inhibition of the PI3K/mTOR pathway, the same investigators subsequently evaluated a triplet regimen of letrozole, abemaciclib, and metformin [42]. The nonrandomized phase II study enrolled 25 patients with ER-positive EC, 84% of whom had NSMP tumors; the ORR was 32% and median PFS was 19.4 months. Exploratory molecular analyses suggested less benefit in tumors with *RB1* or *CCNE1* alterations and greater activity in *CTNNB1*-mutated tumors. The small sample, single-arm design, and multiple subgroup comparisons preclude treatment-selection claims.

#### 7.3.3. XPO1 Inhibition

XPO1 inhibition provides a biologic rationale for study in *TP53*-wild-type EC because nuclear export blockade may retain tumor-suppressor proteins in the nucleus [43]. Exploratory analyses of the SIENDO trial suggested prolonged PFS with selinexor maintenance in *TP53*-wild-type endometrial cancer. In the updated analysis, median PFS was 28.4 months with selinexor versus 5.2 months with placebo (HR, 0.44; 95% CI, 0.27–0.73); in the *TP53*-wild-type/pMMR subgroup, median PFS was 39.5 versus 4.9 months (HR, 0.36; 95% CI, 0.19–0.71) [44]. Because molecularly defined NSMP tumors are generally pMMR and p53 wild-type after exclusion of *POLE*-mutated and MMR-deficient tumors, this subgroup substantially overlaps with, but is not equivalent to, the NSMP population. Accordingly, the SIENDO findings may provide indirect, hypothesis-generating evidence that a subset of NSMP tumors could be sensitive to XPO1 inhibition, but they cannot be directly extrapolated to molecularly confirmed NSMP disease.

The subsequent phase III XPORT-EC-042 trial [45], which prospectively selected patients with TP53-wild-type endometrial cancer, did not meet its primary PFS endpoint. According to company-reported topline results, median PFS in the modified intention-to-treat population was 12.75 months with selinexor and 7.43 months with placebo (HR, 0.76; 95% CI, 0.51–1.12) [46]. Thus, although the earlier SIENDO signal remains biologically relevant to NSMP because of the substantial molecular overlap between *TP53*-wild-type/pMMR and NSMP disease, current evidence is insufficient to establish selinexor maintenance as an NSMP-specific therapeutic strategy. Prospective studies incorporating complete molecular classification, including *POLE*, MMR, and p53 status, are required to determine whether molecularly confirmed NSMP tumors—and potentially specific NSMP subsets—derive clinically meaningful benefit from XPO1 inhibition.

## 8. Summary and Future Outlook

NSMP EC is a heterogeneous molecular category whose clinical behavior is shaped by established clinicopathologic factors and a range of candidate molecular and immune markers. Grade, histotype, ER status, FIGO stage, and LVSI currently have the clearest clinical relevance for prognostic refinement. *CTNNB1* and *ARID1A* alterations, L1CAM expression, and spatial immune phenotypes may add information in selected cohorts, but assay definitions, adjusted effect estimates, and external validation remain inconsistent.

The three-layer framework presented here is intended to distinguish established classification from clinicopathologic refinement and exploratory biomarkers. It is not a validated scoring system, does not assign treatment, and should not be used to infer predictive value from prognostic associations. Future studies should prospectively confirm NSMP status, predefine biomarker thresholds, report treatment interactions, and evaluate incremental discrimination, calibration, clinical utility, feasibility, and cost-effectiveness. These steps are necessary before candidate refiners can support routine surgical or systemic treatment decisions.

## Figures and Tables

**Table 1:** Evidence Level and Current Clinical Role of Prognostic Refiners in NSMP Endometrial Carcinoma.

Prognostic Refiner	Key Evidence in NSMP EC	Evidence Level *	Current Clinical Role
FIGO stage, histotype, grade, and LVSI	Consistently associated with outcome and incorporated into current risk-group assignment [13,16].	High	Established components of routine prognostic assessment and adjuvant-treatment stratification.
ER expression	Large molecular cohorts support prognostic refinement using ER with grade; the 2025 ESGO–ESTRO–ESP guidelines use a 10% cutoff [13,14,15,16].	Moderate–high	Clinically applicable prognostic refiner; should not be used alone as a treatment-predictive biomarker.
PR expression	PR positivity is associated with favorable outcomes, but its independent value beyond ER and grade is inconsistent [13,17].	Moderate	Adjunct to ER and grade, rather than a standalone classifier.
CTNNB1 exon 3 mutation	Associated with recurrence in retrospective studies and incorporated into the integrated profile evaluated in PORTEC-4a [18,19,20,21,22,23].	Moderate	Emerging component of integrated profiling; independent prognostic and predictive value remains unconfirmed.
ARID1A alteration	Several cohorts reported adverse associations, but pooled evidence is inconsistent; mutation and protein loss are not interchangeable [17,24,25,26].	Low–moderate	Investigational prognostic refiner; not recommended for treatment selection.
L1CAM overexpression (>10%)	Associated with recurrence and mortality in retrospective NSMP cohorts and incorporated into the PORTEC-4a integrated profile [23,26,27,28].	Moderate	Emerging adverse prognostic refiner; routine IHC testing is feasible, but clinical utility requires further validation.
Spatial immune phenotype	Inflamed, immune-desert, and immune-excluded phenotypes show different survival outcomes in retrospective NSMP cohorts [12,29].	Low	Research-level prognostic marker; no established treatment-predictive role.
HLA-I loss	Associated with recurrence in a small retrospective cohort, with substantial statistical uncertainty [30].	Very low	Hypothesis-generating marker of immune escape; research use only.
Other genomic or transcriptomic features	KRAS, FGFR2, PI3K-pathway alterations, 1q gain, and interferon-response signatures remain incompletely validated [10,11,12].	Very low	Biological characterization and hypothesis generation only.

Table note: * Evidence levels are qualitative assessments based on study design, consistency, external validation, and incorporation into guidelines or prospective strategies; they are not formal GRADE ratings. Prognostic associations should not be interpreted as evidence of treatment-predictive value. Abbreviations: EC, endometrial carcinoma; ER, estrogen receptor; FIGO, International Federation of Gynecology and Obstetrics; HLA-I, human leukocyte antigen class I; IHC, immunohistochemistry; LVSI, lymphovascular space invasion; NSMP, no specific molecular profile; PR, progesterone receptor.

## Data Availability

No new data were created or analyzed in this study. Data sharing is not applicable to this article.
